# Mr. Southams, on Vaccine Inoculation

**Published:** 1806-11

**Authors:** John Southams

**Affiliations:** Buckingham


### *To the Editors of the Medical and Physical Journal.*

GENTLEMEN,

IN the 88th No. of the Medical and Physical Journal, page 545, Dr. Walker has written, "A surgeon from Buckingham happening to call, on my writing this, mentions a case to me where the inoculation was resisted till the twenty-ninth * time, when a perfect cow-pock was produced;" and in the Medical and Physical Journal for the present month the following remark appears by John Ring, in reply to Dr. Walker: "Dr. Walker sets but little value on the test of inoculation with vaccine virus, and tells us, on the authority of a surgeon from Buckingham, that in one case it did not succeed till the twenty-ninth time. This, however, must have been the fault of the inoculator, or of the matter." As I am the person alluded to, permit me to offer a few words in explanation of the above case. Last winter I received a message from the late William Fermor, Esq.† of Tusmore, a gentleman well known to Dr. Jenner, and I apprehend, also to J. R. as he sent him his different publications on the subject of vaccination, desiring I would come to his house and inoculate two female servants, who had been inoculated before by different practitioners, without success. I went accordingly; and, after I had inoculated one of them, she informed me it was the twenty-fifth time of her being inoculated. As it did not take effect in a few days, I again inoculated her, each time with dry vaccine virus, of the goodness of which I had no reason to doubt, having other patients at the same time who were successfully inoculated with it. As this inoculation proved unsuccessful, I took a patient with me to Tusmore, and inoculated her again (the twenty-

---

* Dr. Walker appears to have made a mistake in the number; it should have been twenty-seventh time, but it is not very material.

† Author of Reflections on the Cow-pox, 1800

twenty-seventh time) with fluid virus, taken immediately from the arm, which succeeded, and produced a perfect vaccine pustule. That the matter was not always in fault, even in the first twenty-four inoculations, I think, may be fairly presumed, as W. F. informed me, some of the matter was procured by him from Dr. Jenner; therefore, according to J. R's. assertion, *the fault must have been in the inoculator*; a conclusion, I think, he is not warranted in making; in my opinion it might have failed from some other cause, and, in support of that opinion, I beg leave to adduce the very respectable authority of Dr. Willan, who, in his Treatise of Vaccine Inoculation, observes, "In the infinite diversity of human constitutions, there may be some which are neither susceptible of the vaccine disease nor the small-pox; others which are susceptible of the former and not of the latter, or vice versa; and others which are susceptible of both at the same time; or, to a certain degree at separate times." The repeated failure in this case was probably owing to some peculiar idiosyncrasy of the constitution, and not as J. R. asserts, in such unqualified terms, merely to the want of skill in the practitioners, or to not having used good matter. It were to be wished that J. R. would abstain from such hasty assertions, and I am concerned to add, in some instances, though not in the present, intemperate language, which too frequently occur in his various writings in favour of vaccination. The truth surely does not stand in need of abuse for its support; fortiter in re, suaviter in modo, I beg leave to recommend to his attention, when he writes again on this subject, for it appears doubtful, if he has not on some occasions, by the language he has adopted, hurt the cause he meant to serve.

I am, &c.

*Buckingham,*

7th of 10th Month, 1806. JOHN SOUTHAMS.

